# First detection of foot-and-mouth disease virus O/Ind-2001d in Vietnam

**DOI:** 10.1371/journal.pone.0177361

**Published:** 2017-06-09

**Authors:** Le T. Vu, Ngo T. Long, Barbara Brito, Carolina Stenfeldt, Nguyen T. Phuong, Bui H. Hoang, Steven J. Pauszek, Ethan J. Hartwig, George R. Smoliga, Pham P. Vu, Le T. V. Quang, Vo V. Hung, Nguyen D. Tho, Pham V. Dong, Phan Q. Minh, Miranda Bertram, Ian H. Fish, Luis L. Rodriguez, Do H. Dung, Jonathan Arzt

**Affiliations:** 1 Regional Animal Health Office No. 6, Department of Animal Health, Ministry of Agriculture and Rural Development, Ho Chi Minh City, Vietnam; 2 Foreign Animal Disease Research Unit, Plum Island Animal Disease Center, ARS, USDA, Orient Point, NY, United States of America; 3 Oak Ridge Institute for Science and Education, PIADC Research Participation Program, Oak Ridge, TN, United States of America; 4 Department of Animal Health, Ministry of Agriculture and Rural Development, Hanoi, Vietnam; Oklahoma State University, UNITED STATES

## Abstract

In recent years, foot-and-mouth disease virus (FMDV) serotype O, topotype Middle East-South Asia (ME-SA), lineage Ind-2001d has spread from the Indian subcontinent to the Middle East, North Africa, and Southeast Asia. In the current report, we describe the first detection of this lineage in Vietnam in May, 2015 in Đắk Nông province. Three subsequent outbreaks caused by genetically related viruses occurred between May–October, 2015 after which the virus was not detected in clinical outbreaks for at least 15 subsequent months. The observed outbreaks affected (in chronological order): cattle in Đắk Nông province, pigs in Đắk Lắk province and Đắk Nông province, and cattle in Ninh Thuận province. The clinical syndromes associated with these outbreaks were consistent with typical FMD in the affected species. Overall attack rate on affected premises was 0.85 in pigs and 0.93 in cattle over the course of the outbreak. Amongst 378 pigs at risk on affected premises, 85 pigs died during the outbreaks; there were no deaths among cattle. The manner in which FMDV/O/ME-SA/Ind-2001d was introduced into Vietnam remains undetermined; however, movement of live cattle is the suspected route. This incursion has substantial implications for epidemiology and control of FMD in Southeast Asia.

## Introduction

Foot-and-mouth disease (FMD) is one of the most important diseases of livestock worldwide, and is endemic in several countries in Asia and Africa [1, 2]. FMD affects animal production at various levels including large commercial operations, individual household subsistence units, and countries’ access to international markets [3]. Within the seven serotypes of FMD virus (FMDV), the distribution of phylogenetically related viral strains and lineages demonstrates that distinct geographical regions share the same viral lineages and that the spread of those lineages beyond their defined regions only occurs on limited occasions [4]. However, there are known exceptions to this trend including the intercontinental spread of FMDV serotype O topotype Middle East-South Asia (ME-SA) lineage PanAsia (O/ME-SA/PanAsia). This virus was first described in the 1980s in the Indian subcontinent and subsequently disseminated widely throughout all Southern Asia, the Middle East, and East and Southeast Asia, with some incursions into South Africa and European countries [5]. In Asia there are three defined virus pools: Pool 1 includes FMDV strains circulating within East Asia and Southeast Asia, Pool 2 includes strains from Southern Asia or the Indian subcontinent (excluding Pakistan), and Pool 3 includes viruses found within the Middle East and West Eurasia [6–8].

Incidence and serotype-specificity of FMDV varies across East and Southeast Asian countries. Vietnam and surrounding countries have high reported disease incidence throughout the year [8]. Three different lineages of serotype O have been circulating in Vietnam during recent years; O/CATHAY, O/ME-SA/PanAsia, and O/SEA/Mya-98 [9, 10]. Additionally, serotype A lineage SEA-97 is endemic in Vietnam, while serotype Asia 1 has not been detected in Vietnam since 2007 [2].

The O/ME-SA/Ind-2001 lineage of FMDV was detected and first defined in 2001 [11] and has become the dominant lineage of serotype O within the Indian subcontinent since 2008 [12]. Other FMDV strains collected before the initial characterization of the lineage (between 1995–2002) in Jordan, Israel, Bahrain, Kuwait, Saudi Arabia, the United Arab Emirates (UAE), Oman, and the Palestinian Autonomous Territories were later classified within the Ind-2001 lineage [13].

From 2008–2009, there were isolated introductions of FMDV O/ME-SA/Ind-2001 into UAE and Iran [14]. There were no further (known) incursions of this lineage outside the Indian subcontinent until 2013, when outbreaks in Libya and Saudi Arabia were reported [13, 15]. Additional outbreaks in Sri Lanka caused by this virus were reported in 2013 and 2014 [16]. In 2014 and 2015 O/ME-SA/Ind-2001 caused several outbreaks in Morocco, Algeria, and Tunisia, as well as in the UAE and Bahrain [13, 17, 18].

The FMDV O/ME-SA/Ind-2001 lineage has been further classified as Ind-2001a, b, c and d sublineages based upon phylogenetic analyses. All outbreaks outside of the Indian subcontinent that have involved the O/ME-SA/Ind-2001 lineage since 2013 have been caused by the sublineage Ind-2001d [12]. In 2015, this lineage was detected for the first time in Southeast Asia in cases reported from Vietnam and Laos [17]. Subsequent outbreaks of O/ME-SA/Ind-2001 lineage occurred in Myanmar in 2015 and 2016 [19].

In Vietnam, cattle and buffalo are commonly vaccinated against FMDV serotype O twice annually with varying vaccine formulations, based upon current knowledge of circulating strains. Population-level coverage of vaccination, and thus herd immunity, is regionally variable. Pigs and small ruminants are typically not vaccinated. When outbreaks occur, suppressive vaccination is often deployed to affected and surrounding premises. Additional outbreak response practices include regional restriction of animal movement and slaughter, disinfection of premises, and establishment of temporary quarantine posts for enforcement until 21 days from the last clinical case.

The purpose of the current report is to document and provide basic descriptive characterization of the first known incursion of FMDV O/ME-SA/Ind-2001d sublineage into Vietnam. This information is critical for consideration of FMDV surveillance and control programs in Vietnam, throughout Southeast Asia, and globally.

## Materials and methods

### Permissions and ethics

The field outbreak investigations described herein were conducted by federal staff of the Regional Animal Health Office (RAHO) 6, Department of Animal Health (DAH) and provincial Sub-DAH offices in Vietnam as part of their official duties. All cases described herein occurred spontaneously in domestic livestock with no experimental inoculation or treatment of live animals. No animals were euthanized for the purpose of this study. Sample collection was performed as part of routine field outbreak investigations; samples were subsequently compiled for the sake of the current investigation. Therefore, ethics approval was not required for the work presented herein.

### Field investigations

Field teams were deployed to outbreak sites within 24–48 hours of notification from regional sources. Upon arrival at outbreak premises, interviews of herd managers and animal owners were performed. All animals on affected premises were examined and epithelial samples were collected for virus characterization. Distinct FMDV isolates were obtained from five distinct samples of the multiple field specimens collected.

For each outbreak premise, “attack rate” was defined as: total number of new cases / population at risk over the course of the outbreak. Similarly, “Case fatality proportion” was defined as total number of deaths / number of diseased animals over the course of the outbreak.

### Detection of FMDV

Upon report of clinical cases of vesicular disease, provincial field veterinarians collected samples of vesicular tissues, which were transported on cold packs to the RAHO 6 facility for analysis. Upon arrival, samples were immediately processed and analyzed by virus isolation and subsequent antigen ELISA for serotype confirmation.

### Virus isolation

Virus isolation was performed following a protocol previously described [20], with minor modifications. In brief, approximately 0.5–1 g of tissue samples were mechanically disrupted and resuspended in minimal essential media (Gibco-Invitrogen, Carlsbad, CA) containing 25 mM HEPES (Gibco-Invitrogen, Carlsbad, CA). Samples were clarified by centrifugation and 250 μl of sample suspensions were inoculated onto BHK-21 cell monolayers. After 1 h of adsorption, 5 ml of media with 1% serum were added to the flasks. The occurrence of cytopathic effect was observed within 48–72 hours post inoculation.

### FMDV antigen ELISA

Clarified virus isolation supernatants were analyzed by FMDV serotype O antigen ELISA (kit number R5605, The Pirbright Institute, UK) per manufacturer’s recommended procedure. This kit is based on an indirect sandwich ELISA technique [21, 22]. In brief, rabbit antisera specific for FMDV serotype O was adsorbed to polystyrene microwell plates. Coated plates were washed in phosphate-buffered saline and 50 μl of clarified virus isolation supernatant or diluted control reagents (provided in kit) were added to each well. All samples were analyzed in duplicate. Plates were incubated for 1 hour at room temperature and were subsequently washed in phosphate-buffered saline. Detection antibody consisting of guinea pig antisera with the same specificity as the rabbit antisera used for trapping was added to the wells and plates were incubated for 1 hour at 35^°^C. After an additional wash step, horseradish peroxidase-conjugated rabbit anti-guinea pig immunoglobulin was added to wells and plates were incubated at 35^°^C for 45 minutes. After washing, substrate/chromogen solution consisting of ortho-phenylenediamine (3.3mM) and hydrogen peroxide (4.4mM) were added to the wells. The color reaction was stopped by addition of 1.25 M sulphuric acid after 15 minutes of incubation. Optical density values were read at 492 nm. Mean OD values >0.1 above background values were considered positive.

### Sequence acquisition and analysis

Subsequent to confirmation of presence of FMDV by virus isolation and antigen ELISA, RNA extraction and RT-PCR were conducted as previously described [5]. In brief, RNA was extracted from tissue suspensions using silica membrane RNeasy spin columns (Qiagen, catalog number 74104) according to the manufacturer’s instructions. Three primer combinations were used for the RT-PCR of serotype O isolates: FMDV-ARS4-F (ACCAACCTCCTTGATGTGGCT) /EUR-2B52R (GACATGTCCTCCTGCATCTGGTTGAT), O-1C244F (GCAGCAAAACACATGTCAAACACCTT) /EUR-2B52R, and O-1C272F (TBGCRGNCTYGCCCAGTACTAC) /EUR-2B52R. The following thermal profile was used: 42°C for 30 min for reverse transcription; 94°C for 5 min for denaturing; 35 cycles of 94°C for 60 s for denaturing; 60°C for 60 s for primer annealing; and 72°C for 90 s for extension; followed by a final extension of 72°C for 5 min. RT-PCR amplicons were visualized on an agarose gel and purified. The purified FMDV amplicons were processed through the 454 sequencing pipeline in three main steps: (1) Rapid Library Preparation (Roche Diagnostics; GS FLX Titanium Rapid Library Preparation Kit; Cat. No. 05608228001), (2) emPCR amplification (Roche Diagnostics; GS emPCR Kit III Cat. No. 04 891 392 001), and (3) sequencing on Genome Sequencer FLX (Roche Diagnostics), as previously described [23]. The FMDV sequences from Vietnam which are described herein have been submitted to GenBank (Table 1).

**Table 1:** Outbreaks of FMDV-O in Vietnam during May–October 2015.

Out-break	Date (2015)	Location	Virus Detected (Genbank#)	No. Farms	Species	No. Animals Affected	Total No. Animals	Attack Rate	Case Fatality Proportion	Vaccine
1	May 26	Đắk Nông	O/Ind-2001d (KY399464)	2	Cattle	12*	15*	80%*	0*	None^a^
2	Sept 10	Đắk Lắk	O/Ind-2001d (KY399465)	1	Pig	45	50	90%	16%	None^a^
3	Sept 28	Đắk Nông	O/Ind-2001d (KY399466)	1	Pig	306	328	93%	25%	O^b^
4	Oct 20	Ninh Thuận	O/Ind-2001d (KY399468)	17	Cattle	5*	5*	100%*	0*	O^b^

*Number of animals reported from the index farm only.

^a^Cattle on surrounding farms had been recently vaccinated with FMDV-O.

^b^Suppressive vaccination with FMDV-O was administered to surrounding cattle and buffalo.

### Phylogenetic analysis

A total of 99 FMDV reference sequences from ME-SA topotype, most belonging to the O/ME-SA/Ind-2001 lineages, as well as the sequences collected from the outbreaks described herein, were used to reconstruct a phylogeny using Bayesian phylogenetic methods. The analysis was carried out in BEAST 1.8.2 using a lognormal uncorrelated relaxed clock and a Bayesian skyline tree prior [24] with 200,000,000 iterations. Mixing and convergence was assessed using Tracer 1.6 [25] making sure that all parameters reached >200 effective sample size. The maximum clade credibility tree was annotated and graphed in Figtree 1.4 [26].

## Results

### Field investigations

**Fig 1:**
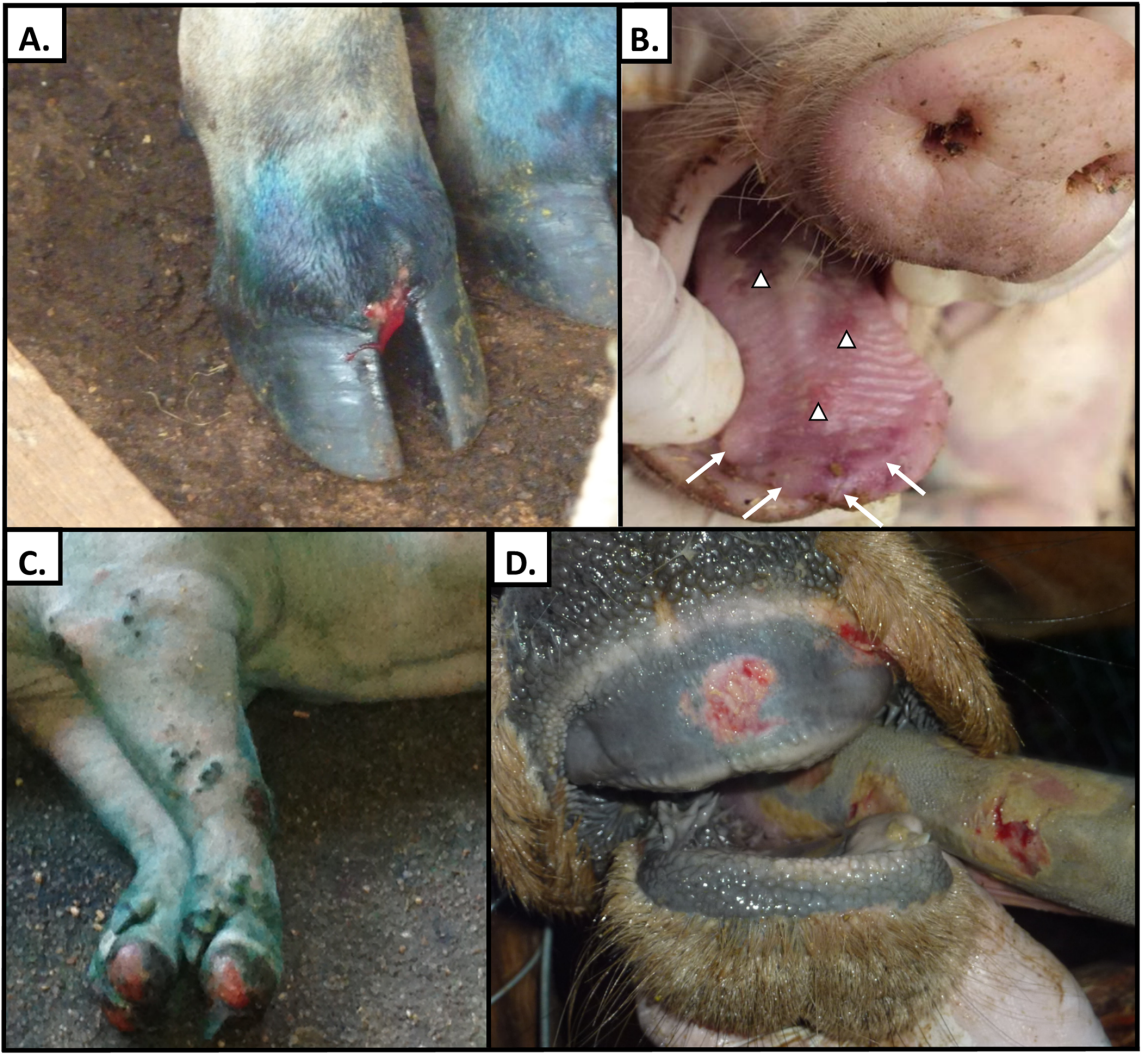
Clinical lesions during outbreaks of FMDV O/Ind-2001d in Vietnam. **A)** Outbreak 1, interdigital cleft lesion, front hoof of a cow. The vesicle has ruptured and there is mild hemorrhage (lesion age is estimated as 2–3 days after clinical onset). **B)** Outbreak 3, intact vesicular lesions (arrows) and sloughed epithelium (arrow heads), tongue of a pig. Some vesicles are still intact suggesting lesion age of approximately 1–2 days after clinical onset). **C)** Outbreak 3, sloughed hooves on front feet of pig from same farm as animal shown in panel B. The sloughing and granulation tissues suggests these lesions are approximately 4–5 days after clinical onset. Green coloration from animal-marking paint. **D)** Outbreak 4, dental pad erosion and sloughed epithelium on tongue of a cow in Ninh Thuận province. Early re-epithelialization and yellow discoloration from secondary bacterial contamination suggests these lesions are approximately 2–3 days after clinical onset.

Outbreak 1 was reported to RAHO 6 on May 26^th^, 2015 as acute and rapid onset of vesicular disease in cattle on a subsistence farm in Đắk Nông province (Table 1). A single sample of vesicular epithelium was submitted to RAHO 6 and confirmed to be FMDV serotype O by virus isolation and antigen ELISA. On May 28^th^, 2015, a field outbreak investigation team was deployed to the field site. The owner reported having recently purchased 14 cattle from Nghệ An province. Over the course of the outbreak, 12/15 cattle (80%) on this farm developed clinical signs of FMD (Fig 1). During the outbreak, the owner sold 3 of the affected cattle to a neighbor, whereby the outbreak spread to a second premise which was not further investigated due to limited resources in the field. As cattle in the region had recently been vaccinated, no suppressive vaccination was administered to surrounding farms. The outbreak did not spread further.

Outbreak 2 was reported to RAHO 6 on September 10^th^, 2015 as acute and rapid onset of vesicular disease in pigs in Đắk Lắk province and was confirmed to be FMDV serotype O by antigen ELISA. The outbreak investigation team was deployed the following day and determined that 45/50 pigs (90%) had clinical signs of FMD. Over the course of the outbreak, 7 pigs in the 12 week-old cohort died. Some pigs were observed to collapse and rapidly die with no prodromal signs of cardiac involvement. As cattle in the region had recently been vaccinated, no suppressive vaccination was administered to surrounding farms. The outbreak did not spread further.

Outbreak 3 was reported to RAHO 6 on September 28^th^, 2015 as acute and rapid onset of febrile vesicular disease in pigs at a high intensity piggery in Đắk Nông province. The cause of the syndrome was determined to be FMDV serotype O by virus isolation and antigen ELISA. Across all age groups, 306/328 (93%) of pigs were affected. Overall case fatality proportion was 25% (78/306) with pigs within 3 age strata dying: Piglet (<8 weeks; 13%), Piglet (8–10 weeks; 20%), Fattening (>16 weeks; 45%). No breeding sows died during the outbreak despite 100% attack rate in this group. Suppressive vaccination (FMDV-O) was delivered to cattle and buffalo on surrounding farms.

Outbreak 4 was reported to RAHO 6 as vesicular disease with lameness in cattle in Ninh Thuận province on October 20^th^, 2015. On October 22^nd^, 2015 FMDV-O was confirmed by virus isolation and antigen ELISA. As of that date, 5/5 (100%) of cattle at the Outbreak 4 index premise were affected and there were unconfirmed reports of additional cases at nearby farms. Suppressive vaccination with FMDV-O monovalent vaccine was immediately initiated in cattle and buffalo throughout the region. Additional clinical cases continued for approximately 14 days affecting at least 17 premises. The precise extent of this outbreak is not known as the speed and extent of spread surpassed the reserve capacity of field veterinarians. This fact, along with the likelihood of under-reporting, confound precise accounting of affected premises and incidence.

### Sequence acquisition and phylogenetic analysis

**Fig 2:**
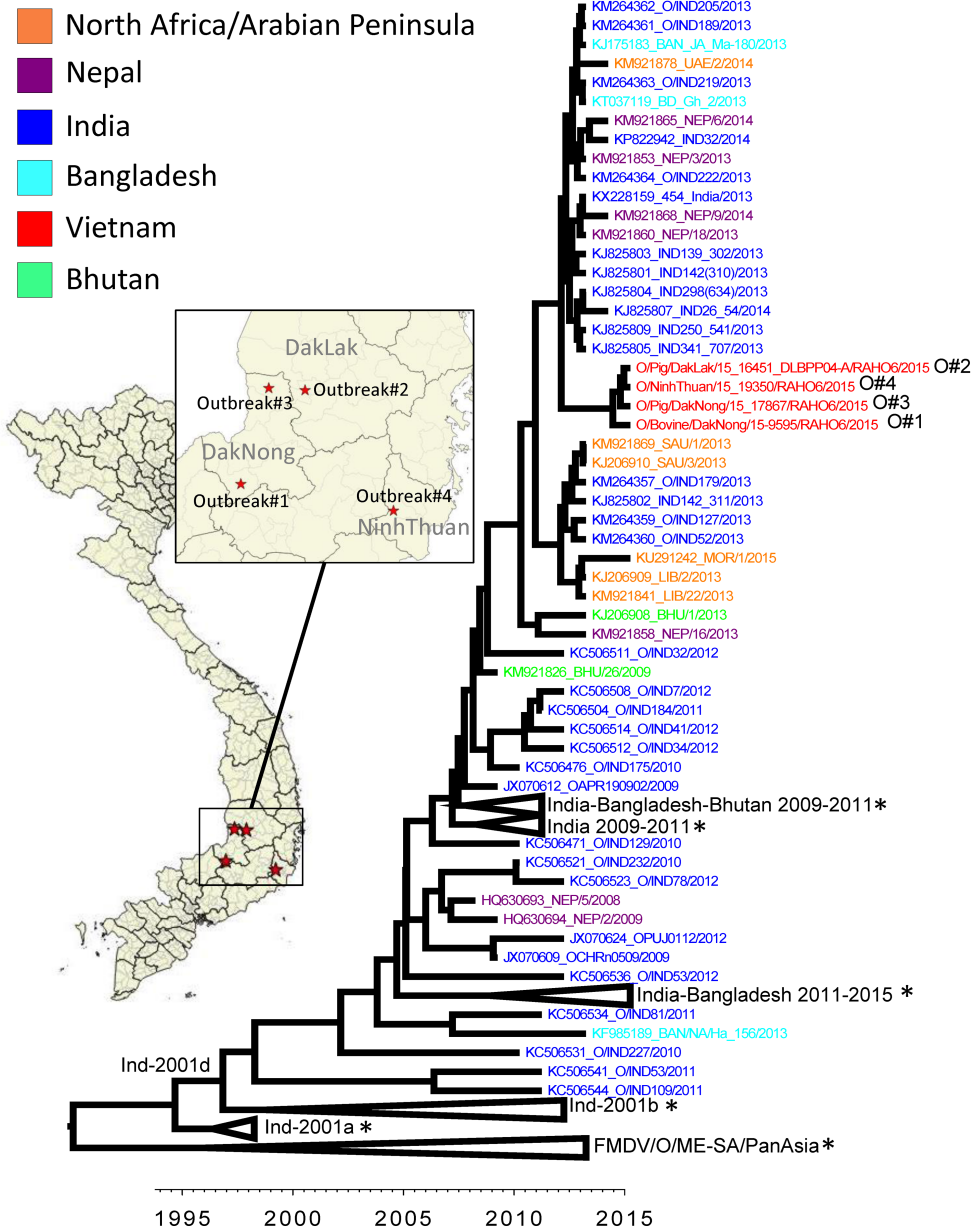
Maximum clade credibility tree depicting the phylogeny of FMDV serotype O ME-SA India-2001. Names of the viruses collected in Vietnam are highlighted in red. Viruses from Vietnam are closely related to other Ind-2001 collected in India, Nepal and Bangladesh collected in 2013 and 2014. O#1, #2, #3, and #4 correspond to outbreak numbers 1–4 described in text and Table 1. Collapsed clades (*) are expanded in full detail in S1 Fig.

The phylogenetic tree reconstructed using FMDV VP1 protein coding segment reference sequences demonstrated that all serotype O viruses collected from the 4 outbreaks belonged to the O/ME-SA/Ind-2001d sublineage (Fig 2, S1 Fig). The estimated year to the most recent common ancestor (MRCA) for the Vietnamese viruses was 2014 (95% high posterior density–HPD = 2014–2015). These viruses formed a clade most closely related to O/ME-SA/Ind-2001 viruses previously reported from India, Nepal, Bangladesh and the UAE in 2013–2014, and time to MRCA of the Vietnamese viruses and the closest reference viruses was estimated at 2012 (95%HPD 2011–2012). The second closest cluster that included sequences from Saudi Arabia, India, Libya, collected in 2013, as well as a sequence collected from Morocco in 2015 had a MRCA estimated at 2011 (95%HPD 2010–2012) (Fig 2).

Sequences collected from the 4 outbreaks in Vietnam were highly similar, differing by 1–3 and 0–2 nucleotides and amino acids, respectively, in their VP1 coding segment. Nucleotide and amino acid differences between the first Vietnamese outbreak (O/Bovine/DakNong/15-9595/RAHO6/2015) and selected sequences from the closest genetic cluster (Fig 2) KM921878 UAE/2/2014, KJ825804 IND298(634)/2013 were 12 nucleotides (1 amino acid) and 11 nucleotides (2 amino acids), respectively (not shown).

The mean substitution rate computed for the Ind-2001 lineage was 5.02 x10^-3^ (95% HPD = 3.83x10^-3^, 6.25x10^-3^) substitutions per site per year in the VP1 protein-coding region, similar to previous estimates of Ind-2001 and PanAsia lineages estimates [12, 27].

## Discussion

In this report, we have described the first detected outbreaks caused by FMDV O/ME-SA/Ind-2001 lineage in Vietnam. The specific sources of these outbreaks remain undetermined. However, the purported transfer of cattle from Nghệ An to Đắk Nông just before the first outbreak is suggestive of the source of incursion. Nghệ An borders Laos where outbreaks of FMDV/Ind-2001d had been reported in June 2015 [28]. Although temporal and geographic associations between the outbreaks in Vietnam and Laos suggest that these events are epidemiologically related, the specific source and manner of introduction of FMDV/Ind-2001d to Vietnam remains undetermined.

The clinical, epidemiological, and pathological presentations of cattle and pigs infected with these strains of FMDV/Ind-2001d were consistent with typical descriptions of FMD [29–31]. Although lesions have been described associated with abortions due to FMDV/Ind-2001d [32], to our knowledge, no previous publication has demonstrated or described characteristics of lesions in adult animals infected with viruses of this sublineage. Sheep and goats were not observed to be affected in any of the outbreaks despite large numbers of these species present within the affected regions. The lack of observation of clinical disease in unvaccinated goats and sheep in close proximity to affected cattle and pigs suggests that small ruminants may be unlikely to become infected or clinically affected by these viruses. However, this negative finding is insufficient to make definitive conclusions about host range or species-specific clinical presentation. Such hypotheses would be more definitively addressed by active surveillance of small ruminants in the field or controlled exposure experiments in a containment laboratory setting.

There has been no additional detection of this lineage in Vietnam subsequent to the outbreaks reported herein. The lack of further evidence of circulation or reintroduction of the O/ME-SA/Ind-2001d lineage in Vietnam suggests that the outbreaks reported herein may represent one or more isolated introductions with limited expansion. However, subclinical infections and under-reporting of outbreaks commonly occur such that O/ME-SA/Ind-2001 may be more prevalent than indicated by reported cases.

In 2016, the O/ME-SA/Ind-2001d lineage was reported in Myanmar associated with an apparently separate introduction of this specific lineage, without relation to the viruses found in 2015 in Laos and Vietnam [19]. Additionally, samples collected recently in 2016 in Thailand were also classified as FMDV O/ME-SA/Ind-2001d [16]. These recent outbreaks and continued detection of O/ME-SA/Ind-2001d in previously unaffected geographical regions suggests continued incursions of this virus should be expected in Vietnam and other countries.

Limited spread of the reported outbreaks also suggests that FMDV vaccines currently used in Vietnam may be at least partially protective against O/ME-SA/Ind-2001d. Antigenic characterization of viruses of this lineage has predicted sufficient cross-reactivity with the antigen O/IND/R2/75 currently used in the Indian vaccines [12, 33]. However, antigenic matching of recently detected O/ME-SA/Ind-2001 viruses to viruses used for vaccine production for use in Southeast Asia is critical to ensure adequate protection.

Substantial movements of large ruminants occur from the Indian Subcontinent into Southeast Asia in association with trade [34, 35]. However, even though the O/ME-SA/Ind-2001 virus has been the dominant FMDV lineage in the Indian Subcontinent since 2008, this FMDV lineage was never detected in Southeast Asia before 2015. It is possible that undetected clinical and subclinical incursions may have occurred on previous occasions. Ongoing retrospective and prospective genetic characterizations of the lineage may clarify this possibility.

The outbreaks reported herein represent the first confirmed incursion of FMDV/O/ME-SA/Ind-2001 into Southeast Asia. It is still uncertain if this lineage will become a dominant strain within Southeast Asia. Continued monitoring of the varying, ongoing trends of endemic FMDV in Southeast Asia will ultimately elucidate the significance of this lineage for regional control and eradication of FMD.

## Supporting information

S1 Fig Maximum clade credibility tree depicting the phylogeny of FMDV serotype O ME-SA India-2001. The tree indicates all sequences used for phylogenetic reconstruction, and the 95% high posterior density of the nodes. This figure shows the details of the clades collapsed in Fig 2.
(TIFF)
