# Supplementary figures and images for: First detection of foot-and-mouth disease virus O/Ind-2001d in Vietnam
Source: PLoS One. 2017 Jun 9;12(6):e0177361. doi: 10.1371/journal.pone.0177361 (PMC5466432; doi:10.1371/journal.pone.0177361)

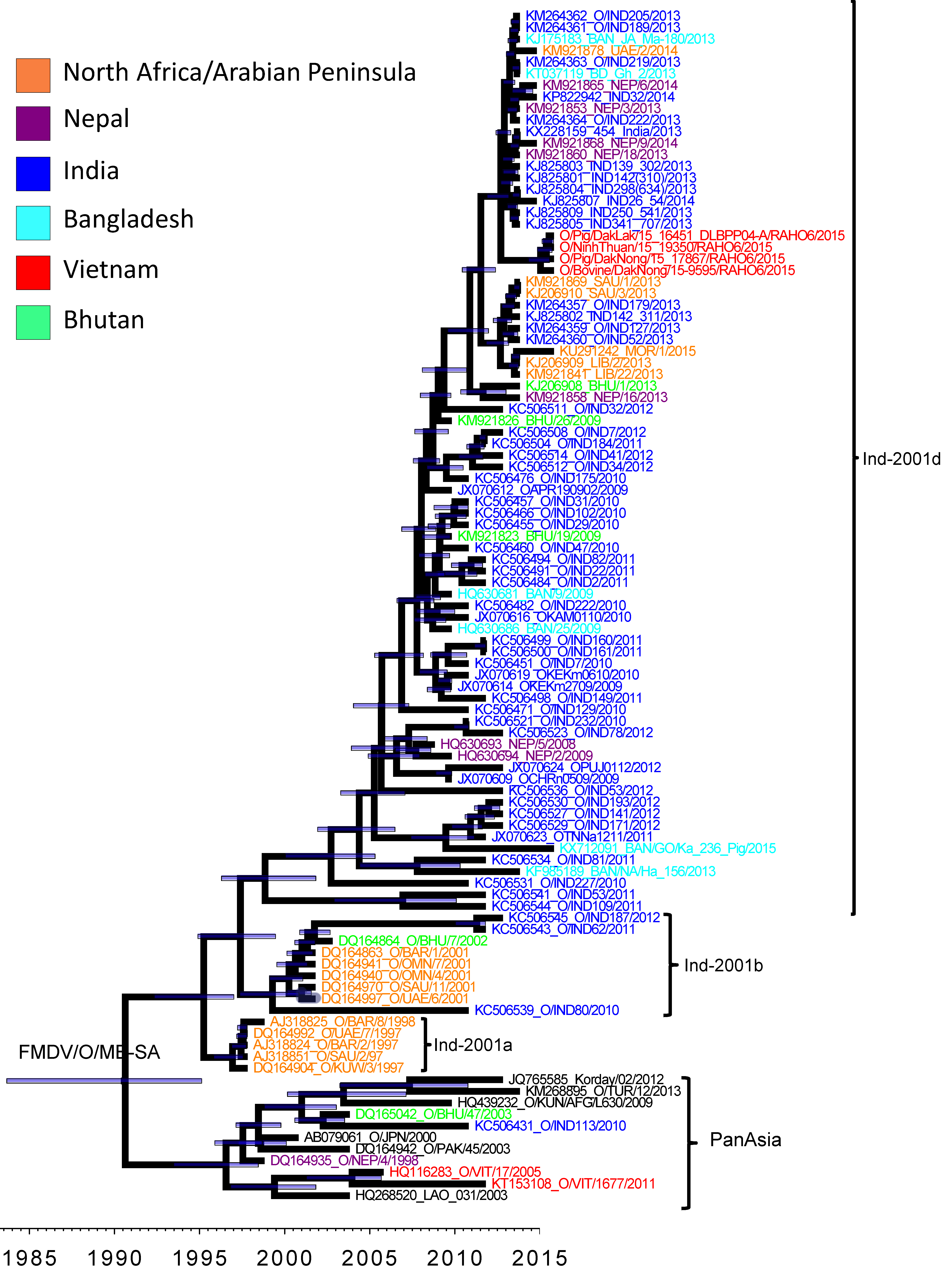

Supplement: S1 Fig — The tree indicates all sequences used for phylogenetic reconstruction, and the 95% high posterior density of the nodes. This figure shows the details of the clades collapsed in Fig 2. (TIFF) [file pone.0177361.s001.tiff]
